# Unexpected Temperature Behavior of Polyethylene Glycol Spacers in Copolymer Dendrimers in Chloroform

**DOI:** 10.1038/srep24270

**Published:** 2016-04-07

**Authors:** Denis A. Markelov, Vladimir V. Matveev, Petri Ingman, Marianna N. Nikolaeva, Anastasia V. Penkova, Erkki Lahderanta, Natalia I. Boiko, Vladimir I. Chizhik

**Affiliations:** 1 St. Petersburg State University, 7/9 Universitetskaya nab., St. Petersburg, 199034, Russia; 2 St. Petersburg National Research University of Information Technologies, Mechanics and Optics, Kronverkskiy pr. 49, St. Petersburg, 197101, Russia; 3 Instrument Centre, Department of Chemistry, University of Turku, Vatselankatu 2, FI-20014, Turku, Finland; 4 Institute of Macromolecular Compounds, Russian Academy of Sciences, Bolshoi Prospect 31, V.O., St. Petersburg, 199004, Russia; 5 Laboratory of Physics, Lappeenranta University of Technology, Box 20, 53851, Lappeenranta, Finland; 6 Faculty of Chemistry, Moscow State University, Leninskie gory, Moscow, 119991, Russia

## Abstract

We have studied copolymer dendrimer structure: carbosilane dendrimers with terminal phenylbenzoate mesogenic groups attached by poly(ethylene) glycol (PEG) spacers. In this system PEG spacers are additional tuning to usual copolymer structure: dendrimer with terminal mesogenic groups. The dendrimer macromolecules were investigated in a dilute chloroform solution by ^1^H NMR methods (spectra and relaxations). It was found that the PEG layer in *G *= 5 generations dendrimer is “frozen” at high temperatures (above 260 K), but it unexpectedly becomes “unfrozen” at temperatures below 250 K (i.e., melting when cooling). The transition between these two states occurs within a small temperature range (~10 K). Such a behavior is not observed for smaller dendrimer generations (*G *= 1 and 3). This effect is likely related to the low critical solution temperature (LCST) of PEG and is caused by dendrimer conformations, in which the PEG group concentration in the layer increases with growing *G*. We suppose that the unusual behavior of PEG fragments in dendrimers will be interesting for practical applications such as nanocontainers or nanoreactors.

Copolymer structures are an important research direction in terms of both fundamental investigations and practical applications. The modification of terminal segments is a natural way to obtain copolymer dendrimers. Typically modified terminal segments differ from inner segments in their physical and/or chemical properties, such as hydrophobicity/hydrophilicity, polarity, rigidity, flexibility, and others^1^,^2^,^3^,^4^,^5^,^6^,^7^. The modification of terminal segments significantly expands the possibility of utilizing both the outer shell of the macromolecule and the inner space of dendrimers.

In our recent paper^8^ we reported that copolymer structures allow one to obtain macromolecules with a “hollow” core and a dense outside shell in the double system: dendrimer-solvent. It was established that incompatibility between inner and terminal segments is the necessary condition for the formation of conformations with a “hollow” core. These studies allowed us to correctly interpret the computer simulation results^9^ and NMR experimental data^10^ for carbosilane dendrimers with terminal mesogenic groups.

The purpose of this paper is to demonstrate the additional properties of dendrimer systems in which a “hollow” core can form^8^,^9^,^10^. As we suppose that under certain conditions polyethylene glycol (PEG) moieties, added to the carbosilane dendrimer with mesogenic groups, can play the role of a “lock” for a substance encapsulated within the dendrimer core. Note that PEG fragments in other types of dendrimers are successfully utilized in biological applications (for instance, see the recent review^11^ by *Thakur et al.*).

The present study concerns carbosilane dendrimers with terminal mesogenic groups attached by PEG spacers (in our previous studies^9^,^10^ aliphatic spacers were used). These dendrimers are copolymer macromolecules due to the difference between the nonpolar dendrimer core and polar terminal groups. The properties of a single dendrimer macromolecule in the dilute chloroform solution are investigated. In our research we use NMR spectra and relaxation. This choice is due to the fact that the NMR technique allows one to “look inside” a dendrimer and to investigate individual parts of a macromolecule (see review^6^ by *Hu et al.* and, for instance, recent refs 12, 13, 14, 15, 16, 17).

The investigated systems are dilute chloroform solutions of carbosilane dendrimers of the *G *= 1, 3, 5 generations (briefly, G1, G3, and G5) with terminal mesogenic groups. The terminal segments are phenylbenzoate mesogenic groups connected by oligo(ethylene glycol) spacers (PEG-BUT or TG1). Additionally we present experimental NMR data for the G5 dendrimer with terminal chiral ethyl-L-lactate-containing mesogenic groups (Und-BPL or TG2). The structural formulas of the studied dendrimers are shown in Fig. 1. The synthesis of studied macromolecules was described in refs 18, 19, 20. The hydrodynamic radius of the G5 dendrimer should be smaller than 6 nm^21^. Concentrations of dendrimers in the solvent were 1.1 wt.% for G1TG1, 0.8 wt.% for G3TG1, and 0.4 wt.% for G5TG1. Note that the deuterated chloroform used contains a small amount of water (0.1 wt.%).

The measurements were carried out at 400 MHz using a BRUKER AVANCE 400 spectrometer. The ^1^H NMR spectra were obtained with the number of accumulations between 8 and 64 depending on dendrimer concentrations. The residual proton signal of deuterated chloroform with a chemical shift (*δ*) 7.26 ppm was used as the internal reference for the calibration of chemical shifts *δ* of spectral lines. No temperature correction was made for the reference *δ* (CНCl_3_). The proton spin-lattice relaxation times, *T*_1H_, were measured using the conventional inversion-recovery *π*-*t*_*d*_-*π*/2 pulse sequence with 6 *μ*s duration of *π*/2 pulse, 14 scans for each *T*_1H_ measurement, and 9s recycle delay time between scans. The latter was longer than any measured 5*T*_1H_. The temperature dependences were obtained in a temperature range limited by the room temperature and the freezing point of the solvent (298 K–218 K). It is more convenient in the discussion to use the inverse value of *T*_1H_, i.e., the spin-lattice relaxation rate *R*_1H_* *= 1/ *T*_1H_.

Figure 2(a–c) shows the ^1^H NMR spectra of the dendrimers in the range of 0 to 5 ppm at room temperature (298 K). The full spectra of studied systems with the identification of spectral peaks in accordance with refs 10,22. are shown in Supplementary Information. Here we consider the selected spectral lines, which demonstrate the observed effect in a higher degree, with special numbering of these lines by *α, β*, γ in ascending order of chemical shifts (see Fig. 2). The peak *α* corresponds to the inner Si-CH_3_ groups, peak *β* represents the signal from the external Si-CH_3_ groups, which are the connectors between the dendrimer core and PEG spacer. Further, this group will be called the terminal CH_3_ group of the dendrimer core. The peaks *γ, γ*′, and *γ*″, corresponding to the PEG spacer, and peak *ε*, corresponding to the group CH_2_-O of the tail of terminal segments, are located in the range from 3 to 5 ppm.

We observed a significant difference between the NMR spectra of low generations and G5TG1. At room temperature G1TG1 and G3TG1 have highly resolved standard spectra (see also ref.^10^.) and they remain almost unchanged when the temperature decreases. In the case of the G5TG1 dendrimer all peaks in the NMR spectrum are strongly broaden at room temperature (Fig. 2c). Moreover, the peaks, corresponding to PEG spacer groups, are nearly undistinguishable in the spectrum (Fig. 3a). This result reflects the low mobility of the molecular groups in the G5TG1 dendrimer and freezing conformational mobility of PEG spacers. However, due to the rotation of the dendrimer as a whole PEG spacers cannot be a completely immobile like in the solid state. The width of a peak should be about 300–400 Hz if the simplest (rough) estimation of *τ*_*rot*_via the Stokes-Einstein formula is used: in chloroform *τ*_*rot*_ ~ 10^−7^ s for the particle with a radius smaller than 6 nm. The width of the peak *γ* is wider than 200 Hz, i.e. of the same order. When the temperature decreases to 250 K the peaks of the PEG spacer groups become recognizable and the further decrease in temperature barely changes the NMR spectrum (Fig. 3b). Moreover, the width of the peak *γ* for the G5 dendrimer becomes similar to ones for G1 and G3 dendrimers. These results demonstrate an unusual effect, namely, an increase of the mobility of the PEG spacers with temperature decrease.

Thus, we have to conclude that the G5TG1 dendrimer becomes “unfrozen” with decreasing temperatures. This is an unexpected effect. Therefore, we have carried out the spin-lattice ^1^H NMR relaxation measurements (*R*_1_* *= 1/*T*_1_) of individual groups in the investigated objects. This allows us to estimate the mobility of separate molecular groups in more details.

The experimental results for the spin-lattice relaxation rates of protons for a few molecular groups (*α, β*, and *γ*) are presented in Fig. 4a–c. Note that the *R*_1_ values for CH_2_-O groups of the PEG spacer (*γ*-group) of the G5TG1 dendrimer have a sharp increase under temperature growing in the same range where the peculiarities in NMR spectra were detected. The similar but weaker effect takes place for CH_3_-groups (*α*- and *β*-groups).

In order to obtain an additional information on the observed effect we tried to analyze our relaxation data in details. Temperature dependences of spin-lattice relaxation rates in objects with high molecular mobility are usually interpreted with BPP–Solomon theory^23^,^24^,^25^,^26^,^27^, which in the case of the dipole-dipole relaxation mechanism of ^1^H nuclei (protons) gives:


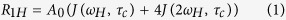


where *J* is the spectral density of the fluctuating interaction, which determines the relaxation process; *τ*_*c*_ is the correlation time, which characterizes the orientational mobility of an observed group; *ω*_*H*_ is the cyclic resonance frequency (2π*f*_0_) for ^1^H nuclei; *A*_0_ is a constant that does not depend on temperature and frequency. For the ^1^H nucleus this constant is given by the expression: 
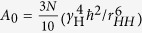
, where *ħ* is the reduced Planck constant (*h*/2*π*); *r*_HH_ is the distance between hydrogen atoms in a group; *γ*_*H*_ is the gyromagnetic ratio for ^1^H nucleus; *N *= 1 and 2 for CH_2_- and CH_3_-groups, respectively. Recent theoretical and experimental studies have shown the applicability of the spin-lattice NMR-relaxation method for the investigation and characterization of the orientational mobility in dendrimer^12^,^15^,^17^. For ^1^H nuclei of dendrimers the spectral density *J* can be described using so-called “free model” approach with two correlation times:^28^


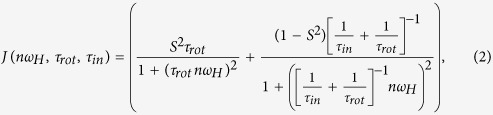


where *τ*_*in*_ and *τ*_*rot*_ are the correlation times which correspond to intrinsic mobility and the rotation of a dendrimer as a whole, respectively; *S* is the generalized order parameter^28^; *n *= 1, 2.

In the case of dendrimers *τ*_*in*_is the characteristic time of the orientational mobility of an individual dendrimer branch and *τ*_*rot*_ is determined by the volume of macromolecules (i.e. strongly depends on *G*)^17^. Figure. 5 reflects different realizations of temperature dependences of *R*_1_ in accordance with Eq. 2, that will be discussed below.

For CH_3_ groups in dendrimer solutions the conditions *S*^2^ ≪ 1 and *τ*_*in*_ ≪* τ*_*rot*_is realized, and for the explanation of the results (Fig. 4a,b) we could employ the simplified one-correlation time approximation of the spectral density, i.e. we neglected the first component in Eq. (2). In general, the contribution from dendrimer rotation as a whole decreases with the topological distance from the dendrimer center and becomes practically equal to zero for terminal segments at the high operating frequency (400 MHz) in the considered temperature diapason.

Figure 4a,b shows the temperature dependence *R*_1H_ for the internal (*α*) and external (*β*) CH_3_ groups of dendrimers. It is evident that the *R*_1H_ values for the G1TG1 and G3TG1 are practically identical. Moreover, such behavior is typical for carbosilane dendrimers with terminal mesogenic groups where the PEG fragment is absent. For example, these dependences coincide with the *R*_1H_ temperature dependence for G5TG2 (without PEG moiety, see Fig. 4a,b). In the case of G5TG1 the *R*_1H_ dependence has a complicated character (Fig. 4c). At low temperatures (right part of the dependences), the relaxation rates *R*_1H_ for both the inner and terminal CH_3_ groups of G5TG1 have the similar behavior as for G1TG1, G3TG1 and G5TG2. At higher temperatures there is a “jump”, and then the *R*_1H_ function remains nearly constant with increasing temperature. This behavior can be explained on the basis of the scenario showed in Fig. 5a. Remind that the NMR spectra provided the information on the rather sharp decrease in the mobility of PEG spacers with an increase in temperature. If there is a structural reorganization in a system, then in the case of slower molecular mobility in the new structure, the theoretical dependence should be shifted to the left side (red curves in Fig. 5a). The bold blue arrow explains the behavior of the experimental dependences for G5TG1 (compare with Fig. 4a,b). A similar (but much stronger) effect is observed for the temperature dependence of *R*_1H_, corresponding to the groups *γ* of the PEG spacer (Fig. 4c). The temperature dependence for G5TG1 at low temperatures (the right side of the dependence) coincides with data for G3TG1. At temperatures near 250 K the sharp “jump” in the values of *R*_1H_ for G5TG1 is observed. The explanation of the effect can be found in Fig. 5b, which repeats Fig. 5a but the shift of the relaxation rates is bigger due to the drastic inhibition of the molecular mobility in the PEG spacer. Note that the temperature, at which the “jumps” in the *R*_1H_ dependences occur, corresponds to the temperature changes in the NMR spectrum of G5TG1 when the peaks of the PEG spacer are detectable/not detectable. That is why there is no a possibility to measure *R*_*1*_ values at high temperatures.

The effect discussed testifies to the sharp slowing down in the orientational mobility of groups of the PEG spacers.

Additionally, note two concomitant features of the data presented in Fig. 4c:

1. The bend (or poorly defined maximum) of the *R*_1H_ temperature dependence for the G3TG1 dendrimer is detected in the range of “freezing” of the PEG fragments of G5TG1. Apparently, the similar effect manifests in G3TG1 but in a weaker form.
2. The *R*_1H_ values for G1TG1 are greater than for G3TG1. For the explanation of this fact we suggest to take into account that the first generation of i.e. the dendrimer does not have a complete dendrimer structure. It seems the contribution of the rotation of a dendrimer molecule as a whole plays the more important role for PEG groups in G1TG1because in this case *τ*_*rot*_ is the shortest rotational time for the studied dendrimers. Thus, the relation *τ*_*rot *_~ *τ*_*in*_ can be expected for G1TG1 that leads to the superposition of two maxima in the *R*_1_ temperature dependence which, in turn, results in increasing the overall maximum (see Fig. 5c).

Thus, we can conclude that the data of NMR spectroscopy and NMR relaxation show the unusual behavior of the G5TG1 dendrimer: (i) the “freezing” of the rotational mobility of the PEG groups (and of the dendrimer segments also) at high temperatures and (ii) the “unfreezing” of the mobility at low temperatures. It is important that the dendrimer is soluble at all temperatures investigated due to the fact that chloroform is a “good” solvent for the rest part of the dendrimer (dendrimer core and mesogenic groups)^10^.

It has previously been shown that carbosilane dendrimers with terminal mesogenic groups possess a “hollow” core and more dense outside layer at room temperature^8^,^9^,^10^. This conformation is formed due to the special location of terminal segments on the periphery. Therefore, in our case it is safe to assume that in the investigated macromolecules there is a spherical layer consisting essentially from PEG spacers. Growing the number of generations leads to an increase in both the concentration of PEG monomer units and the thickness of this layer. It seems that PEG units interact with each other more strongly than with solvent molecules. As a result, this process causes the “freezing” of the PEG layer. The total molecular weight (MW) of all PEG segments in G5TG1 is about 28.000 Da. Even a PEG polymer with significantly higher MW which are usually called poly(ethylene oxide)^29^, dissolves in chloroform at room temperature. We suppose that the discovered effect for PEGs in G5TG1 corresponds to the decreasing of the low critical solution temperature (LCST)^30^,^31^ to 250 K. Unfortunately, we could not find data for LCST of PEG polymer in chloroform solutions. However, LCST for copolymers (but without PEG fragments) in chloroform can be lower than the room temperature^32^.

In the case of water solutions LCST was observed and studied in detail for PEG polymers^33^,^34^,^35^,^36^. The existence of LCST is usually associated with the formation of “PEG–water” hydrogen bonds at temperatures lower than LCST and their destruction at higher temperatures. Moreover, for recently synthesized oligo(ethylene glycol)-based (OEG) dendrimers LCST significantly decreases with the number of generations^37^. For the solution of the G1 OEG dendrimer at 0.25 mg/mL concentration in water, the LCST point was not observed up to 373 K. For the G2, G3, and G4 OEG dendrimers the LCSTs decreases to 304, 297, and 290 K, respectively. As it was recently predicted by molecular dynamics simulation^38^, bifurcated hydrogen bonds between chloroform molecules and PEG groups can exist. Therefore, we suppose that a similar behavior is also possible in our case.

Basing only on our data it is impossible to establish the existence of “PEG–chloroform” hydrogen bonds. However, the presence of water in the CDCl_3_ solvent allowed us to estimate the interaction between water molecules and PEG spacer atoms by investigating the chemical shifts of the water peak in NMR spectra (other parts of the macromolecules are hydrophobic). In chloroform without dendrimers the water chemical shift *δ* increases by only on ≈ 0.2 ppm when the temperature changes from 298 K to 223 K (Fig. 6). Similar dependence is observed for solutions of a dendrimer without PEG units (G5TG2). This is to be expected because in this case, all groups of the dendrimer are hydrophobic and do not interact with the water molecules. In the presence of the hydrophilic PEG dendrimer fragments there is strong displacement of the water peak with the inverse temperature. In the investigated temperature range the increase of *δ* for G1TG1 and G3TG1 is up to 6 ppm and 3 ppm, respectively. This proves that PEG groups interact with water molecules. At the same time, in the case of G5TG1 no interaction between PEG and Н_2_O is observed because the behavior of the water peak is practically the same as in the case of the hydrophobic dendrimer or in the absence of dendrimers in the solvent. Therefore, it can be concluded that the tight package of PEG fragments pushes out the water molecules from this layer.

Thus, we present for first time the evidence that in the dilute chloroform solution at room temperature the PEG groups “gum up” together in G5TG1 which consists of [*G*5 generations carbosilane dendrimer] - [PEG spacers] - [mesogenic groups]. It is shown that the mobility of these groups is very low (nearly comparable to solids). A similar effect does not occur for the structures of smaller generations of dendrimers. After decreasing the temperature to 250 K, the mobility of the PEG groups in G5TG1 becomes similar to one in dendrimers with a smaller number of generations, i.e., the PEG groups begin to “unfreeze”. We suppose that the observed effect is the result of the specific conformation of the dendrimer structure: hollow core - dense peripherals. This structure is caused by the high concentration of terminal segments on the periphery of the macromolecule that, in turn, must lead to the increase in the concentration of PEG groups in the proper layer when the number of generations increases. The growth of PEG concentration in G5TG1 decreases the LCST to a temperature up to 250 K–260 K.

In summary, we suppose that the obtained data can be explained by the following: At temperatures higher than LCST the PEG groups of G5TG1 dendrimers do not form hydrogen bonds with solvent molecules and as a result the chloroform is a “poor” solvent for the groups. However for the dendrimer core and mesogenic groups the chloroform is still a “good” solvent at high temperature and as a result the dendrimer is dissolved. It leads to the segregation of PEG groups in the layer between the dendrimer core and the mesogenic groups. A similar effect is not observed for G1TG1 and G3TG1 because their LCSTs are higher than 320 K. At temperatures lower than LCST the PEG groups of the G5 dendrimer can form hydrogen bonds with solvent. In this case the chloroform is a “good” solvent for PEG groups that leads to the recovery of the mobility of the PEG groups in the G5 dendrimer.

In addition, we would like to note that the usage of the unusual behavior of the PEG layer as a “lock” can be a promising idea. The boundary between the open and closed states of the “lock” is determined by the LCST. The transition between these states occurs in a small temperature range (~10 K). We suppose that this effect can be found for other systems like [dendrimer] - [PEG spacer] - [solvophilic group]. The transition temperature (i.e., LCST) can be regulated by selecting certain dendrimer structural parameters such as the number of generations, the functionality of the core or the branching points, and the length of PEG spacers. Therefore, we believe that the observed effect is of considerable practical interest for both the improvement of existing encapsulation methods and the design of new types of dendrimer nanocontainers or nanoreactors.

## Additional Information

**How to cite this article**: Markelov, D. A. *et al*. Unexpected Temperature Behavior of Polyethylene Glycol Spacers in Copolymer Dendrimers in Chloroform. *Sci. Rep.* **6**, 24270; doi: 10.1038/srep24270 (2016).

## Supplementary Material

Supplementary Information

## Figures and Tables

**Figure 1:**
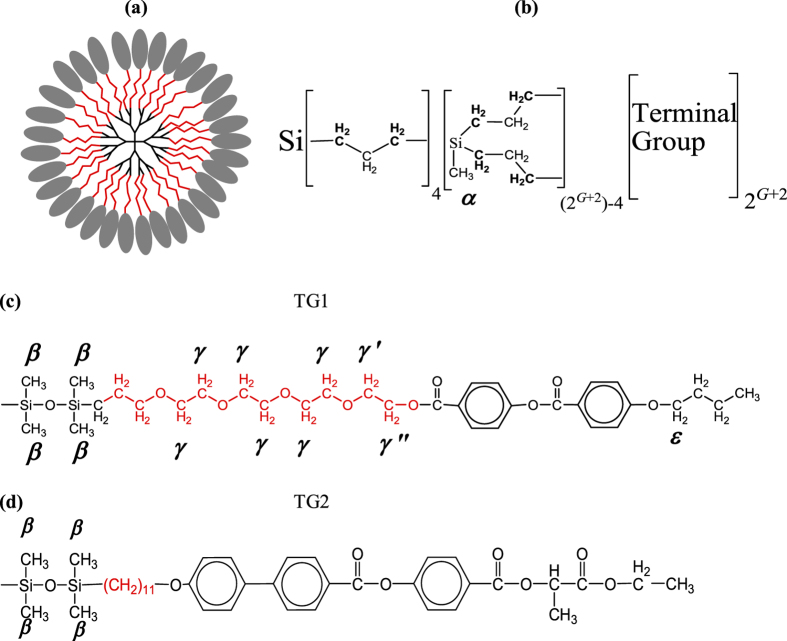
The schematic formula (**a**) of G3TG1 dendrimer with PEG-mesogenic terminal groups and structural formulas (**b**–**d**) of carbosilane dendrimers with terminal mesogenic groups: TG1 is the phenylbenzoate mesogenic group with the oligo(ethylene) glycol fragment (PEG-BUT) and TG2 is the chiral ethyl-L-lactate–containing mesogenic group (Und-BPL). Spacers between the dendrimer core and mesogenic groups are indicated by red. Groups, which are marked by α, β, γ, and ε symbols, give corresponding peaks in NMR spectra (see Fig. 2).

**Figure 2:**
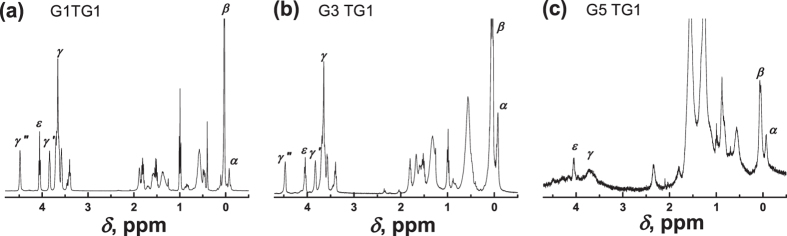
(**a**–**c**) The ^1^H NMR spectra of carbosilane dendrimers with PEG-BUT terminal groups (TG1) in the dilute CDCl_3_ solution at 298 K. The numbering of lines corresponds to Fig. 1.

**Figure 3:**
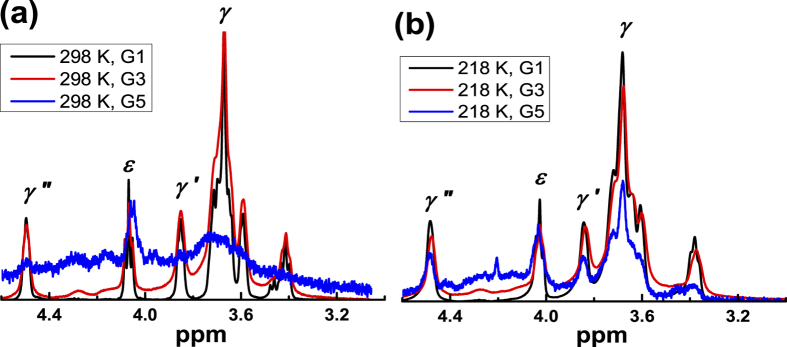
The ^1^H spectra of the PEG fragments of dendrimers at (**a**) 298 K and (**b**) 218 K. The numbering of lines corresponds to Fig. 1.

**Figure 4:**
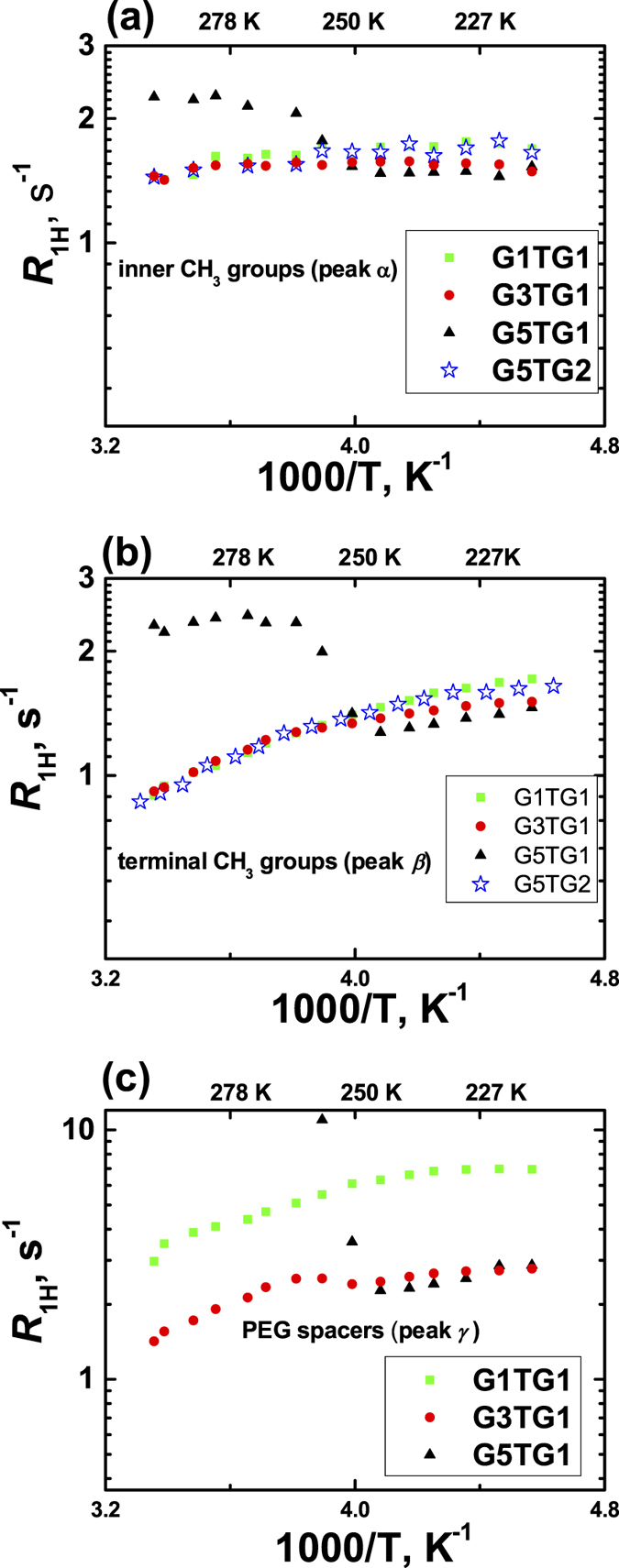
Temperature dependences of R_1H_ for (**a**) inner and (**b**) terminal CH_3_ groups of the dendrimer core; R_1H_ for (**c**) peak γ from PEG spacers.

**Figure 5:**
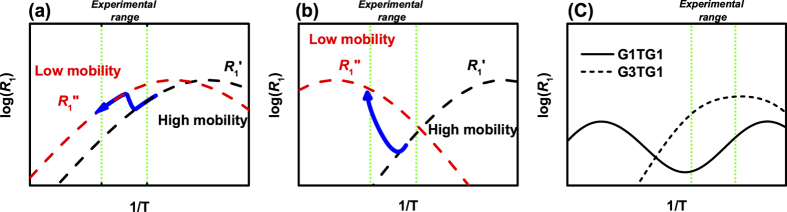
The scenario of temperature dependences of R_1H_ for (**a**) CH_3_ groups (see Fig. 4a,b) and for (**b**) PEG groups (see Fig. 4c) of G5TG1(see explanation in the text). Black and red curves ponds to higher and lower mobility regimes, respectively. Blue arrows show the transition between the regimes. (**c**) The general scenario of temperature dependences of R_1_ for two correlation times (τ_rot_ and τ_in_ see Eq. 2) and S^2^ = 0.5, where a solid and dashed lines correspond to the cases of τ_rot_/τ_in_* *= 100 and 5, respectively. Dotted vertical lines show an experimentally studied temperature range.

**Figure 6:**
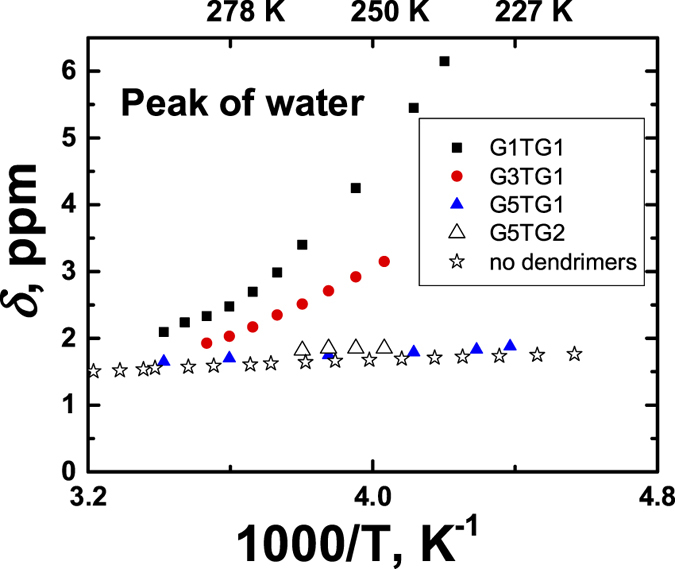
The temperature dependences of water chemical shifts δ in chloroform with and without dendrimers.
