# Supplementary material for: Unexpected Temperature Behavior of Polyethylene Glycol Spacers in Copolymer Dendrimers in Chloroform
Source: Sci Rep. 2016 Apr 7;6:24270. doi: 10.1038/srep24270 (PMC4823654; doi:10.1038/srep24270)
Supplement: Supplementary Information [file srep24270-s1.pdf]

SUPPLEMENTARY INFORMATION

Unexpected Temperature Behavior of Polyethylene Glycol Spacers in Copolymer Dendrimers in Chloroform

Denis A. Markelov,[1,2,*] Vladimir V. Matveev,[1] Petri Ingman,[3] Marianna N. Nikolaeva,[4] Anastasia V. Penkova,[1] Erkki Lahderanta,[5] Natalia I. Boiko,[6] Vladimir I. Chizhik[1]

[1]*St. Petersburg State University, 7/9 Universitetskaya nab., St. Petersburg, 199034 Russia.*

[2]*St. Petersburg National Research University of Information Technologies, Mechanics and Optics, Kronverkskiy pr. 49, St. Petersburg, 197101, Russia*

[3]*Instrument Centre, Department of Chemistry, University of Turku, Vatselankatu 2, FI-20014, Turku, Finland.*

[4]*Institute of Macromolecular Compounds, Russian Academy of Sciences, Bolshoi Prospect 31, V.O., St. Petersburg, 199004, Russia.*

[5]*Laboratory of Physics, Lappeenranta University of Technology, Box 20, 53851, Lappeenranta, Finland.*

[6]*Faculty of Chemistry, Moscow State University, Leninskie gory, Moscow, 119991, Russia.*

1. Structure and NMR spectra of carbosilane dendrimers with terminal phenylbenzoate group connected by oligo(ethylene) glycol (PEG) spacer.

Fig. S1 shows the ^{1}H NMR spectra of the dendrimers at 298 K and 218 K. We identified spectral peaks with different molecular groups of dendrimers using materials of Refs.[10,22]. The numbering of peaks of the NMR spectrum in Fig. S1 corresponds to one of the groups in Fig. S2. Spectra of the studied dendrimers can be divided into three main parts. The peaks of the dendrimer core are mainly located in strong fields (0-2 ppm).

[*] Corresponding Author, Email: markeloved@gmail.com

Accordingly the identification in Ref.[10] peak α corresponds to the inner Si-CH_3 groups. Peak β represents the signal from the external Si-CH_3 groups, which are the connectors between the dendrimer core and the PEG spacer. The inner CH_2 groups contribute peaks to 1 and 3. Peaks 2, 4, and 6 appear in NMR spectrum of mesogenic groups with butyl (BUT) groups (see, for instance, Fig. S7 in "Supplementary Information" of Ref.[22]). It is natural to identify the line with CH_3 groups of BUT groups because this line is narrow and possesses the smallest chemical shift in this groups (~1 ppm). Chemical shifts are used for the recognition of CH_2 groups of BUT tail. we identify the last peak 5 in this region as belonging to the first CH_2 group of PEG spacer (without neighboring oxygen atom).

Peaks, γ, γ', γ'', 7 (corresponding to the PEG spacer), and peak ε, (corresponding to the group CH_2-O of the tail of terminal segments) are located in the range of 3 to 5 ppm. The ^{1}H NMR spectra for the carbosilane dendrimer with the same mesogenic groups (with and without BUT tail), connected by aliphatic spacers, allow one to identify lines ε and γ''. In the case of absence of BUT tails only one peak is observed in the spectrum (~4.3 ppm, see Fig. S6 in "Supplementary Information" of Ref.[22]). Thus, this peak (γ'') corresponds to the CH_2-O-C(O) groups. In our case this peak has higher chemical shift (4.45 ppm) due to the influence of PEG fragment in the studied dendrimer. Second peak in the region appears for the dendrimer structure with BUT tail at 4.05 ppm (see Fig. S7 in "Supplementary Information" of Ref.[22]). Due to this fact, we can accurately identify the peak ε at 4.05 ppm in our case. Other groups of the PEG spacer (7, γ, and γ') we assigned with the structural formula (Fig. S2) in accordance with the increase in chemical shifts.

In the diapason of 6-9 ppm there are a few peaks, corresponding to proton signals of aromatic mesogenic groups. For more details "Supplementary Information" of Ref.[22] can be used.

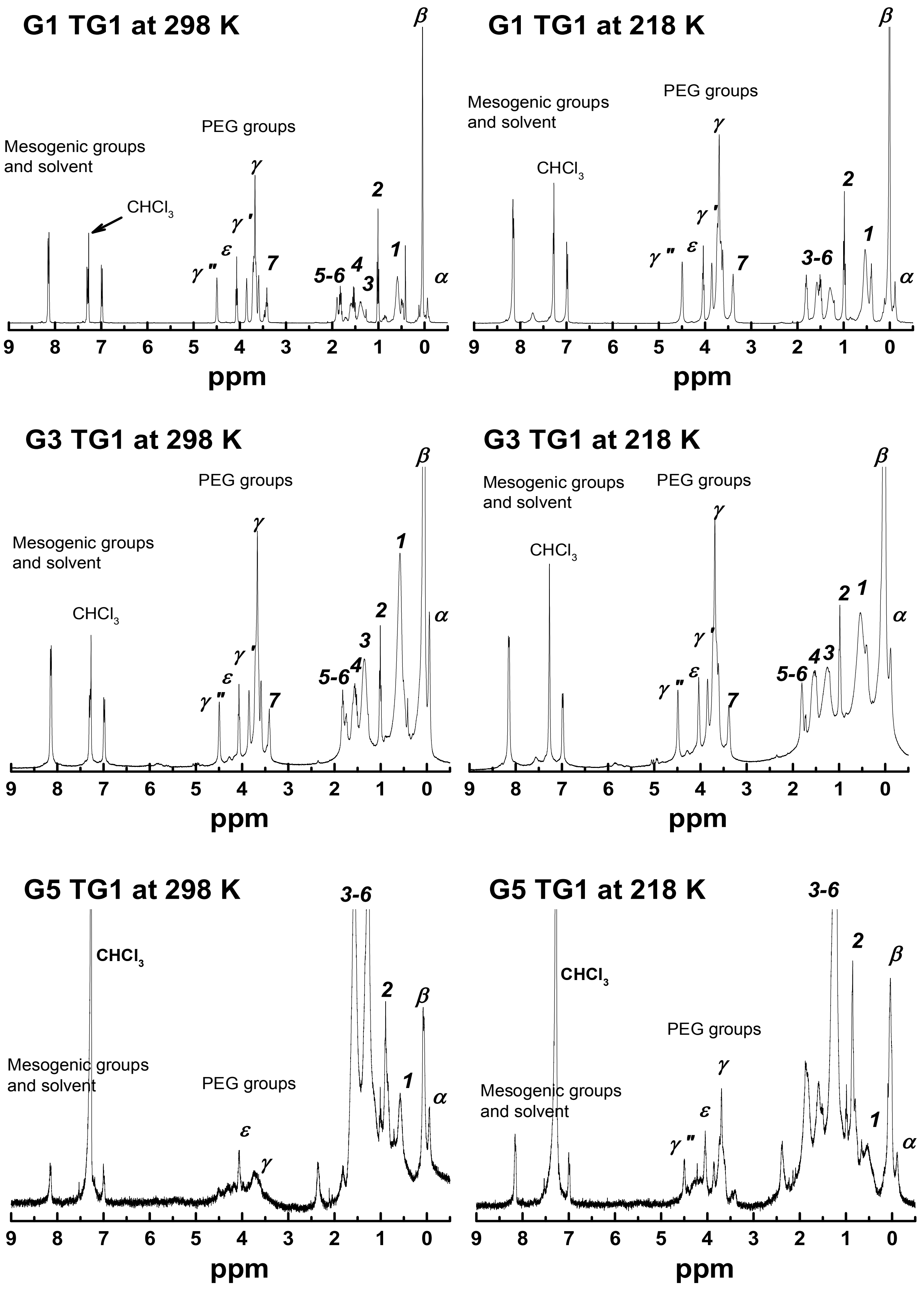

Figure S1. *The ^{1}H NMR spectra of carbosilane dendrimers with PEG-BUT terminal groups (TG1) in the dilute $CDCl_3$ solution at 298 K. The numbering of lines corresponds to Fig. S1.*

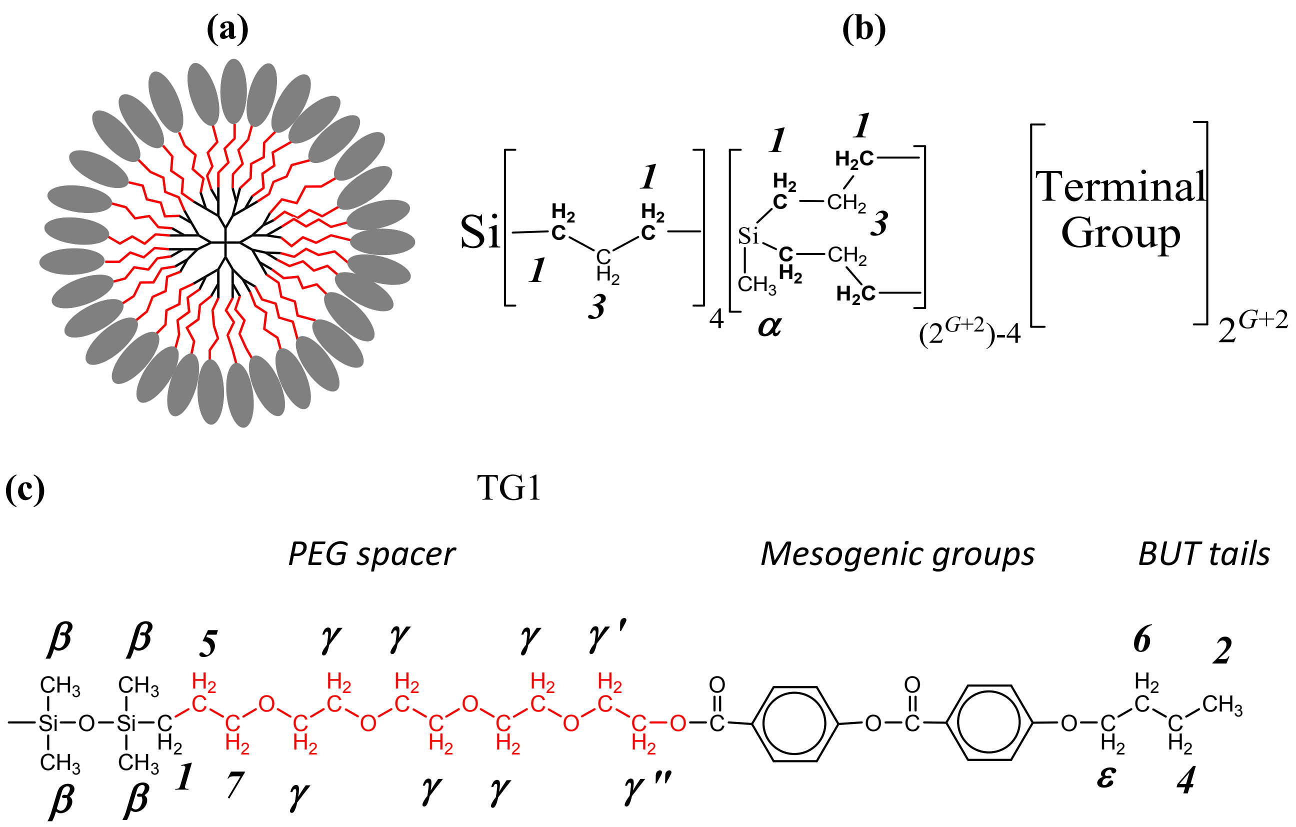

Figure S2. *The Schematic formula (a) of the G3TG1 dendrimer with PEG-mesogenic terminal groups and structural formulas (b)-(c) of carbosilane dendrimers with the terminal phenylbenzoate mesogenic group, TG1 connected by the oligo(ethylene) glycol fragment, PEG. Numbering of groups corresponds to peaks in NMR spectra (see Fig. S1).*

2. Temperature evolution of PEG peaks in G5 TG1

Fig. S3 reflects the final changes of PEG moiety in G5TG1 dendrimer from room temperature to 218 K. At room temperature the spectrum of the groups practically is not resolved in contrast to the spectrum at low temperature.

The temperature evolution of the shape of the peaks is shown in Fig. S4. It can be seen that the changes in the spectrum occurs in a fairly wide range of temperatures (250 K - 260 K).

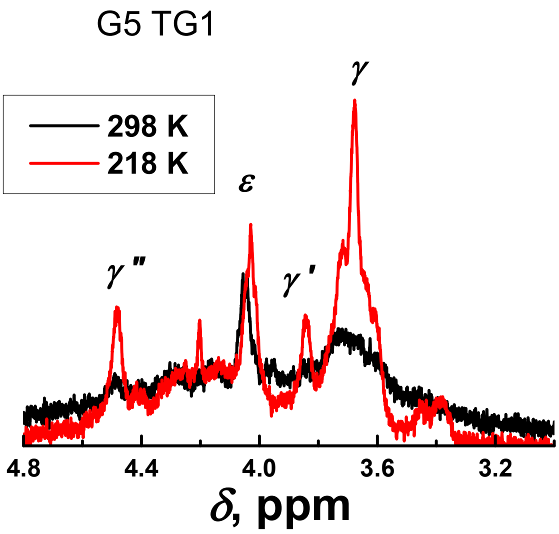

Figure S3. *The ^{1}H spectra of the PEG fragments of the G5TG1 dendrimer at 298 K and 218 K temperatures. The numeration of peaks corresponds to one of the groups in Fig. S2.*

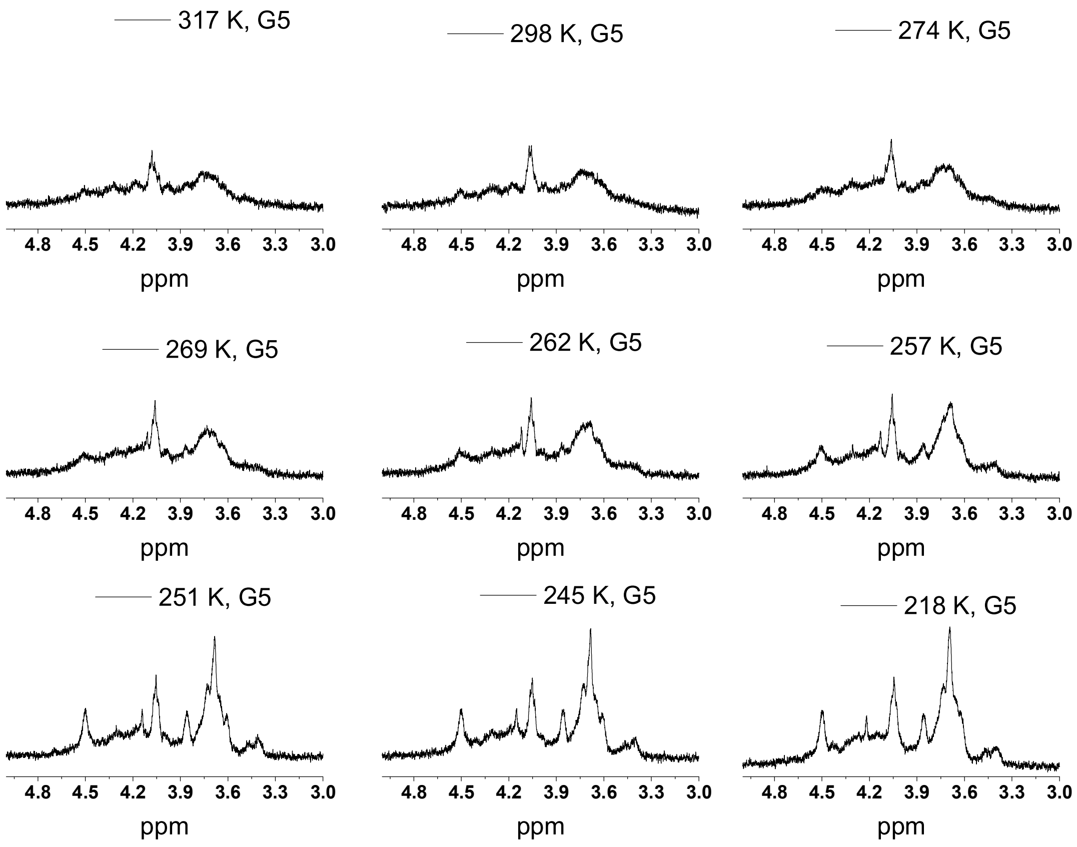

Figure S4. *Temperature evolution of peaks of PEG spacer groups*
